# Endothelial-to-Mesenchymal Transition in Cancer

**DOI:** 10.3389/fcell.2020.00747

**Published:** 2020-08-14

**Authors:** Nicolas Clere, Sarah Renault, Isabelle Corre

**Affiliations:** ^1^Micro and Nanomédecines Translationnelles, Université d’Angers, INSERM UMR U1066, CNRS 6021, Angers, France; ^2^Sarcomes Osseux et Remodelage des Tissus Calcifiés, Université de Nantes, INSERM UMR U1238, Nantes, France

**Keywords:** endothelial, mesenchymal, plasticity, cancer, CAF, metastasis, resistance

## Abstract

Cancer is one of the most important causes of morbidity and mortality worldwide. Tumor cells grow in a complex microenvironment constituted of immune, stromal, and vascular cells that supports growth, angiogenesis, and metastasis. Endothelial cells (ECs) are major components of the vascular microenvironment. These cells have been described for their plasticity and potential to transdifferentiate into mesenchymal cells through a process known as endothelial-to-mesenchymal transition (EndMT). This complex process is controlled by various factors, by which ECs convert into a phenotype characterized by mesenchymal protein expression and motile, contractile morphology. Initially described in normal heart development, EndMT is now identified in several pathologies, and especially in cancer. In this review, we highlight the process of EndMT in the context of cancer and we discuss it as an important adaptive process of the tumor microenvironment that favors tumor growth and dissemination but also resistance to treatment. Thus, we underline targeting of EndMT as a potential therapeutic strategy.

## Introduction

The tumor microenvironment (TME) is a complex network of stromal fibroblastic, immune, and endothelial cells (ECs), embedded in a supportive extracellular matrix. It is now considered as an essential player in cancer biology, participating to tumor progression, metastatic dissemination, and immune surveillance of cancer cells but also to responses to therapies (Maman and Witz, 2018). Furthermore, in response to environmental and oncogenic signals that evolved continuously while tumor growths, the TME appears highly dynamic and plastic (Quail and Joyce, 2013). In this environment, ECs that line microvessels are the key masters of angiogenesis. Indeed, in response to tumor environmental cues, ECs promote the formation of new vessels through proliferation, migration, adhesion, and matrix digestion to support tumor progression and dissemination (Potente et al., 2011). But beside their role in angiogenesis, ECs have been characterized the last decade as capable of an important phenotypic plasticity (Dejana et al., 2017), illustrated by their ability to modify their endothelial phenotype toward a mesenchymal profile. This plasticity, named the endothelial-to-mesenchymal transition (EndMT), was initially described in cardiac embryonic development but has been also highlighted in several postnatal pathologies such as cardiac fibrosis, atherosclerosis, pulmonary hypertension, and vascular calcification (Li et al., 2018a). Endothelial-to-mesenchymal transition appears as essential in these inflammation-associated disorders and may be a key link between endothelial dysfunction and inflammation (Cho et al., 2018). In the context of cancer, the process of EndMT was identified in 2007 in melanoma and pancreatic tumor mouse model (Zeisberg et al., 2007) and critically involved in tumor progression. Since then, increasing evidence has reinforced the relevance of endothelial plasticity through EndMT in cancer biology. This review aims to describe the main features and roles of EndMT in cancer and to highlight its importance in shaping a tumor supportive microenvironment that favors tumor growth, metastatic dissemination, and resistance to treatment. Therefore, targeting EndMT as therapeutic strategy will also be discussed.

## Description

**FIGURE 1:**
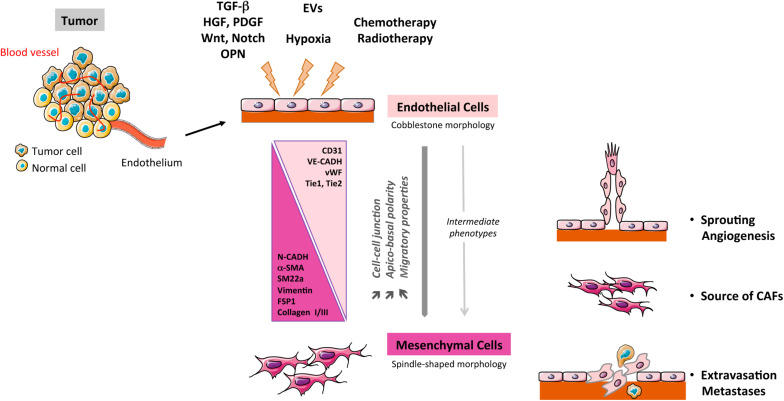
Schematic representation of the EndMT process in cancer. TGF-β, transforming growth factor-β; PDGF, platelet derived growth factor; HGF, hepatocyte growth factor; CAFs, cancer-associated fibroblasts; OPN, osteopontin; EVs: extracellular vesicles.

The EndMT process is characterized as a transdifferentiation program where ECs lose their endothelial characteristics and gain mesenchymal features. This phenotypic switch is characterized by profound morphological, functional and molecular changes. Endothelial cells lose their cellular adhesion and delaminate, reorganize their cytoskeleton that converts their apico-basal polarity toward a front-end back polarity to form spindle-shape cells with enhanced properties of migration. This transition is accompanied with a marked decrease in endothelial markers such as VE-cadherin, CD31, Tie-1, Tie-2, and vWF conjugated to an increased expression of mesenchymal biomarkers such as α-SMA, SM22a, CD44, N-Cadherin, vimentin, COL I/III, and FSP-1 (S100A4) (Piera-Velazquez and Jimenez, 2019) (Figure 1). As described by Dejana et al. (2017), EndMT markers evolve from an early step with partial downregulation of endothelial markers and up-regulation of some early mesenchymal markers (α-SMA, SM22a, and FSP-1) to a later step, characterized by a decline of endothelial markers and up-regulation of mesenchymal markers such as matrix proteins and matrix metalloproteases (MMPs) (Fibronectin, COL I, and MMPs). Initially considered as a complete process of differentiation, EndMT might also be partial in pathophysiological context and in particular in cancer. Intermediate stages of differentiation in tumors-derived ECs have been identified (Xiao et al., 2015) and these tumor ECs display heterogeneity in their potentiality to undergo EndMT. This is consistent with the phenotypic heterogeneity of ECs in tumors (Maishi et al., 2019; Goveia et al., 2020), combined with the diversity of signals emanating from TME, as fine tuning of EndMT appears to be under the control of several factors such as transforming growth factor-β (TGF-β) and basic fibroblast growth factor (bFGF) (Xiao and Dudley, 2017). Moreover, partial EndMT has been assimilated as an initial step of endothelial sprouting in angiogenesis process (Welch-Reardon et al., 2015; Wang et al., 2017). Reversibility of EndMT is barely described in cancer, but a mesenchymal-to-endothelial transition was recently suggested as contributing to Kaposi sarcoma (Li et al., 2018b). Few *in vitro* studies suggest that EndMT reversibility may occur for a short period of time upon exposure to EndMT inducers (Rieder et al., 2011), while prolonged exposure forces mesenchymal differentiated cells to reach a no-return point toward endothelial features (Xiao et al., 2015).

Identifying the process of EndMT in living animals remains challenging. This process is inherently transitional and progressive, rendering detection of mesenchymal cells originating from endothelium difficult unless cells have not terminated their full transition. Therefore, detecting EndMT may be quite often underestimated *in vivo*. Nevertheless, exploration of EndMT has been rendered possible *in vivo* through fate mapping approach in transgenic mice harboring EC-specific lineage tracing, with the limitation that endothelial promoters are rarely totally exclusive to endothelial lineage (Li et al., 2018a). The first *in vivo* evidence of EndMT in a tumor context was shown in subcutaneous melanoma model in Tie-2-Cre x R26TRosa Lox-stop-LacZ-Lox crossed mice (Zeisberg et al., 2007). Since then, fate-mapping tracing has allowed to characterize EndMT in models of lung (Choi et al., 2018), pancreas (Anderberg et al., 2013), and brain (Huang et al., 2016). More importantly, EndMT has now been discovered in tumor specimen of human patients in a number of cancers: colorectal carcinoma (Fan et al., 2018), pancreatic ductal adenocarcinoma (Fan et al., 2019), lung cancer (Choi et al., 2018), or glioblastoma (GBM) (Huang et al., 2016; Table 1).

**TABLE 1:** Inducers and Roles of EndMT in different types of cancer.

Type of cancer	Inducers	Role	Models (Ref)
Melanoma	*nd*	Source of CAFs	Murine model (Zeisberg et al., 2007)
	Tumor-derived TGF-β	Metastasis	Cellular model (Krizbai et al., 2015)
Colorectal cancer	TGF-β1/2	Source of CAFs	Cellular model (Wawro et al., 2018)
	OPN	Source of CAFs	Patients biopsies, cellular model (Fan et al., 2018)
	EVs (mir-92a)	Angiogenesis	Cellular model (Yamada et al., 2019)
Pancreatic cancer	*nd*	Source of CAFs	Patient biopsies, murine model (Zeisberg et al., 2007; Fan et al., 2019)
	Endoglin deficiency	Metastasis	Murine model (Anderberg et al., 2013)
Lung cancer	Radiation	Resistance to radiotherapy	Patients biopsies, murine model (Choi et al., 2018)
	Tumor secretome	Resistance to chemotherapy	Cellular model (Kim et al., 2019)
Glioblastoma	HGF/β-catenin	Resistance to chemotherapy	Patients biopsies, murine model (Huang et al., 2016, 2020)
	PDGF	Resistance to anti-angiogenic agents	Murine model (Liu T. et al., 2018)
Oral squamous carcinoma	Wnt-5b	Lymphangiogenesis	Cellular model (Wang et al., 2017)
Esophageal cancer	TGF-β2-IL1β	Source of CAFs	Patients biopsies, cellular model (Nie et al., 2014)

*OPN, osteopontin; EV, extracellular vesicles; CAFs, cancer-associated fibroblasts; TGF-β, transforming growth factor-β; PDGF, platelet derived growth factor; nd, not determined.*

## Regulation of EndMT

### Major Inducers in Cancer

Members of TGF-β family of proteins are considered today as the major inducers of EndMT, both in physiologic cardiac development (Garside et al., 2013) but also in cancer, as TGF-βs are soluble factors often found overexpressed in tumors (Principe et al., 2014). Therefore, TGF-β1 produced by melanoma tumor cells leads to EndMT (Krizbai et al., 2015). TGF-β2 secreted by tumor in synergy with Interleukin-1β was shown essential to induce EndMT in esophageal carcinoma (Nie et al., 2014). TGF-β2 is also required to promote EndMT in the context of invasive colon carcinoma, *via* a tubulin-β3 dependent mechanism (Wawro et al., 2018). Nevertheless, additional signals to TGF-βs may be required, as highlighted in a 3D spheroid model of lung cancer where EndMT does not rely on TGF-β (Kim et al., 2019). In fact, TGF-β signaling appears insufficient in promoting EndMT in GBM but requires the Notch signaling pathway (Marin-Ramos et al., 2019). Such a crosstalk between Notch and TGF-β participates to the formation of a transient mesenchymal/endothelial niche in a murine breast cancer xenograft model (Ghiabi et al., 2015). In cancer studies, additional soluble factors have been depicted as EndMT inducers. In brain tumor, Hepatocyte growth factor (HGF) (Huang et al., 2016) and platelet-derived growth factor (PDGF) (Liu T. et al., 2018), two growth factors secreted by tumor cells, appear critical to drive EndMT. The bone matrix protein, osteopontin (OPN), through its binding to integrin αvβ3, promotes EndMT through an epigenetic controlled-repression of VE-cadherin in colorectal cancer (Fan et al., 2018). The Wnt signaling pathway through β-catenin/LEF signaling has also be shown to promote EndMT in oral squamous carcinoma (Wang et al., 2017) and very recently in GBM (Huang et al., 2020).

Hypoxia is an important hallmark of the TME and plays a major role in promoting angiogenesis. Nevertheless, hypoxia has also been described as a potent inducer of EndMT, especially in pulmonary arterial hypertension (Suzuki et al., 2018) and in radiation-induced fibrosis (Choi et al., 2015), through hypoxia-inducible factor (HIF)-dependent pathways (Tang et al., 2018; Toullec et al., 2018). Alternatively, hypoxia-induced EndMT in primary and immortalized ECs was shown to involve RhoJ leading to HIF-dependent and trimethylated histone H3K4-dependent pathways (Liu L. et al., 2018). Furthermore, expression of TGF-β, the master inducer of EndMT, may also be regulated by hypoxia (Nishi et al., 2004; Hung et al., 2013). Endothelial-to-mesenchymal transition-relied transcription factors Snail (Xu et al., 2015) and Twist-1 (Mammoto et al., 2018) are also identified as targets of hypoxia. Although particularly relevant in tumors, hypoxia, and HIF signaling remain to be deeper explored in EndMT in cancer.

Responses to cancer treatment have important issues, leading to efficiency but often to resistance. Cancer therapies such as conventional radiotherapies (Mintet et al., 2015; Choi et al., 2018), high-dose radiation therapies (Banerjee et al., 2020) but also chemotherapies (Murugavel et al., 2018) are also currently identified as promoting EndMT in diverse type of tumors, with consequences in tumor response as described below.

### Main Intracellular Signaling Modulators of EndMT

#### Transcription Factors

As mentioned, TGF-β induces EndMT in cancer and involves the Snail family of transcription factors such as Snail, Slug, Twist, and ZEB. These factors are potent transcriptional repressors, notably of endothelial markers VE-cadherin and CD31 (Piera-Velazquez and Jimenez, 2019). A direct regulation of Snail by TGF-β2 has been highlighted in mouse embryonic stem cell-derived ECs (Kokudo et al., 2008) and Medici et al. (2011) described Snail as a master transcription factor of EndMT, induced both by Smad-dependent and PI3K/p38 MAPK-dependent signaling pathways. Importantly, Snail has been identified as a direct target of HIF-1α in hypoxia-induced EndMT (Xu et al., 2015). Furthermore, hypoxia-induced EndMT involves TGF-β-induced phosphorylation of transcription factor Twist-1 (Mammoto et al., 2018) but also increased expression and nuclear translocation of Snail, Slug and Zeb1 (Doerr et al., 2016). TGF-β2 drives also EndMT through a Smad-dependent activation of the myocardin-related transcription factor-A (MRTF-A), in pancreatic MS-1 ECs (Mihira et al., 2012) while in an invasive colorectal cancer microenvironment, TGF-β2-induced EndMT involves MRTF-A and B transcription factors through Smad-independent RhoA pathway (Ciszewski et al., 2017).

#### Epigenetic Regulation

With EndMT characterized as a cellular transdifferentation where gene expression changes are necessary, epigenetics is now considered as a strong regulator of this process (Hulshoff et al., 2018). Furthermore, epigenetic regulation is also determinant for cellular differentiation during cancer pathologies (Petronis, 2010). The global histones methylation of lysine residues has been reported to be associated with EndMT. Thus, EZH2 (enhancer of zeste homolog 2), the major histone methyltransferase that processes the transcriptional repressive trimethylation on lysine 27 of histone H3 (H3K27me3), is down-expressed during EndMT induced by IL-1β and TGF-β2 (Maleszewska et al., 2015). This is associated with reduced H3K27me3 repressive marks at the promoter of the mesenchymal gene *transgelin/SM22*α. Recently, in colorectal cancer, EZH2 was identified as interacting with the transcription factor TCF12, helping this latter to transcriptionally repress *VE-cadherin* gene and thus facilitating EndMT (Fan et al., 2018). Additionally, Jumonji domain-containing protein 2B, JMJD2B, a demethylase of the repressive histone mark H3K9me3, is induced by EndMT-promoting stimuli and is associated to de-repression of genes in EndMT-related genes and pathways (Glaser et al., 2020). Description of EndMT in melanoma intratumoral ECs has recently shed the light on two transcription factors from the ETS family, ERG and Fli-1 (Nagai et al., 2018). These pivotal transcription factors promote expression of EC-specific genes but indirectly repress mesenchymal-related genes through epigenetic regulation by modifying histones methylation/acetylation marks. The dowregulation of ERG and Fli1 has been described in tumor-induced EndMT both *in vitro* and in clinical cancer patients (Nagai et al., 2018), pointing the alleviation of mesenchymal genes repression.

Accumulative evidence highlights that non-coding RNAs including miRNAs and lncRNAs are important epigenetic regulators of EndMT [for review, Hulshoff et al. (2018)]. Depending on their gene targets that mainly belonged to TGF signaling pathways, miRNAs may promote or repress the process of EndMT. The list of miRNAs regulating EndMT is constantly increasing in pathologies (cardiac, fibrosis, and pulmonary hypertension) (Kim, 2018). In malignant disorders, overexpression of miR-302c suppresses EndMT, therefore inhibiting hepatocarcinoma tumor growth (Zhu et al., 2014). A few of lncRNAs have been associated with EndMT. Metastasis-associated lung adenocarcinoma transcript 1 MALAT-1 facilitates EndMT through down-regulation of *Smad3*, *TGFBR2*-targeting miR-145 (Xiang et al., 2017) and recently, a combination of low expression of three lncRNAs (LOC340340, LOC101927256, MNX1-AS1) was proposed as an EndMT index in pancreas adenocarcinoma (Fan et al., 2019).

#### Metabolic Regulation

Activation of the EndMT program has been recently associated with profound metabolic alterations in ECs. Fatty acid oxidation (FAO) was shown to contribute to vessels sprouting by promoting, in angiogenic ECs, a *de novo* nucleotide synthesis for DNA replication for proliferation (Schoors et al., 2015; Eelen et al., 2018). Xiong and colleagues now demonstrate that inhibition of FAO, marked by a decreased expression of mitochondrial enzyme carnitine *O*-palmitoyltransferase 1 (CPT1) and by a fall of acetyl-CoA levels, is critical for TGF-β-induced EndMT (Xiong et al., 2018). Therefore, metabolic plasticity and metabolic transcriptome heterogeneity among normal and tumor ECs, as recently described (Rohlenova et al., 2020), have to be considered as important indicators of endothelial fate, with engagement to EndMT associated with a lower energy-producing metabolism of FAO inhibition.

## Roles of EndMT in Cancer

### Angiogenesis as a Partial EndMT

Angiogenesis is required for an optimal tumor progression (Folkman, 1971) and is initiated by the sprouting of ECs from existent vessels. Sprouting-leader endothelial tip cells lose their apical-basal polarity, weaken their cell-cell interaction, degrade extracellular matrix to become more motile, but never complete their transition as they do maintain some cell-to-cell interactions. Overall, this sprouting resumes EndMT features and may be compared to a partial EndMT (Potenta et al., 2008; Welch-Reardon et al., 2015). Moreover, the master EndMT transcription factors Slug and Snail are found expressed in tumor ECs in several carcinoma (Welch-Reardon et al., 2015), with Slug expression recently associated with angiogenesis and tumor metastasis (Gu et al., 2017). Recently, colon cancer cells, *via* extracellular vesicles, have been shown to induce a partial EndMT associated with angiogenesis, in a miR-92a-dependent inhibition of junctional Claudin-11 and up-regulation of Snail, and vimentin (Yamada et al., 2019). Partial EndMT, stimulated by tumor-secreted Wnt ligand, has also been involved in oral cancer lymphangiogenesis (Wang et al., 2017).

### EndMT as a Source of Cancer-Associated Fibroblasts (CAFs)

CAFs are major components of the TME, contributing to tumorigenesis in multiple ways: cancer cell proliferation, migration, invasion, stemness, mainly through the secretion of cytokines, chemokines, extracellular vesicles, and matrix remodeling [for review, Bu et al. (2019)]. CAFs may originate from several cell types, namely resident fibroblasts, epithelial cells, mesenchymal stromal stem cell from bone marrow, and adipocytes. However, myofibroblastic/mesenchymal cells issued from ECs through EndMT represent also a unique source of CAFs, as initially described in mouse model (Zeisberg et al., 2007). The actual contribution of EndMT in CAFs production is still not fully characterized. Nevertheless, several studies indicate that EndMT-derived cells share functions with CAF-like cells, such as adhesion to collagen and wound healing (Wawro et al., 2018), production of VEGF in esophageal cancer (Nie et al., 2014) but also promotion of angiogenesis, tumor growth and stemness in colorectal cancer (Fan et al., 2018) or driving monocyte M2 polarization in pancreatic cancer (Fan et al., 2019).

### EndMT and Metastasis

One major hallmark of EndMT is endothelial cytoskeletal reorganization through the Rho/ROCK signaling pathway (Mihira et al., 2012; Ciszewski et al., 2017), coupled to the loss of endothelial adhesion molecules (VE-cadherin, claudins). Therefore, EndMT contributes to disruption of the endothelial barrier that may favor the intra- and extravasation tumor cells. Indeed, melanoma cells increase their transendothelial migration when EndMT is induced by tumor secretome (Krizbai et al., 2015). To the same extend, hepatic and lung metastatic seeding is increased in pancreatic cancer mouse model upon endoglin deficiency-induced EndMT (Anderberg et al., 2013). Hence, EndMT by facilitating tumor transendothelial migration can be considered as an essential process for an optimal metastatic dissemination (Gasparics et al., 2016).

### EndMT and Therapeutic Resistance

Vasculature is a double-edge sword in tumor, as it promotes tumor progression through angiogenesis but at the same time it assures delivery of drugs to tumor. Therefore, plasticity and dynamics of the endothelial compartment may impact response to therapies. The role of EndMT in resistance to therapies emerges as a novel field, both in the context of chemo- and radiotherapy. In fact, EndMT confers tumor radioresistance and promotes colorectal tumor growth tumor, by awaking cancer stem cells and stimulating polarization of M2 tumor-associated macrophages TAM (Choi et al., 2018). Resistance to chemotherapies cisplatin and gefitinib in a multicellular lung tumor spheroid model is alleviated when EndMT is reversed, implying EndMT as a resistance factor (Kim et al., 2019). Recently, a robust EndMT induced by HGF/c-Met was described in GBM, resulting in aberrant vasculature and in an increase of tumor cells chemoresistant to temozolomide (Huang et al., 2016), undoubtedly linked to drug delivery failure and hypoxia. The same group documented the induction of resistance of EndMT-issued cells to anti-angiogenic chemotherapies in this brain tumor. Indeed, PDGF-induced EndMT is associated with a decreased expression of VEGFR2, resulting in resistance to anti-VEGF treatment (Liu T. et al., 2018) whereas HGF/β-catenin-induced-EndMT leads to endothelial temozolomide resistance through up-regulation of drug pumping and efflux proteins ABBC1/Multidrug resistance associated protein (MRP-1; Huang et al., 2020). Furthermore, EndMT induces abnormal recruitment of pericytes (Choi et al., 2018) and generates pericyte-like cells abnormally covering the tumor vasculature (Anderberg et al., 2013). Such an aberrant coverage of pericytes is described as a signature of resistance to anti-VEGF anti-angiogenic therapy in two different cancer models [pancreas (Helfrich et al., 2010) and melanoma (Franco et al., 2011)]. Hence, EndMT appears to participate to establish a perivascular niche supportive for resistance in many ways: promotion of hypoxia, failure of drug delivery, vanishing of drug target (VEGFR2), and recruitment of pro-tumoral immune cells. Overall, EndMT has to be considered as a relevant factor of resistance.

## Targeting of EndMT

In light of the overall pro-tumoral effect of EndMT, targeting of this process could represent a therapeutic avenue to treat cancer. Currently, several drugs have been tested as potential EndMT inhibitors. The mTOR inhibitor rapamycin is able to block EndMT, either by preventing EC migration and matrix degradation (Gao et al., 2011) or by lowering TGF-β, TNF-α and VEGF levels (Gonzalez-Mateo et al., 2015). Spironolactone, an aldosterone receptor-blocker, abrogates EndMT in a model of fibrosis, by blocking TGF-β and Notch signaling (Chen et al., 2015). Notably, a derived-form of temozolomide, the standard chemotherapy in GBM, can suppress EndMT in tumor-associated ECs via the same mechanisms of TGF-β and Notch signaling inhibition (Marin-Ramos et al., 2019), supporting the clinical value of this molecule.

An innovative adjuvant strategy could use molecules targeting metabolic pathways. As inhibition of fatty acid oxidation is described to potentiate EndMT (Xiong et al., 2018), one may consider activation of FAO to limit the EndMT process. Indeed, pharmacological approaches to increase FAO are available, mainly through the use of peroxisome proliferator-activated receptor PPAR agonists (Djouadi et al., 2005) or fatty acid synthase FASN inhibitors (Thupari et al., 2004) but are not explored yet in cancer.

Inhibiting kinase signaling engaged by recently identified EndMT-inducing factors such as PDGF or HGF (Huang et al., 2016; Liu T. et al., 2018) would be of interest. For example, Nintedanib, a tyrosine kinase inhibitor of PDGF, FGF, and VEGF, has been shown to ameliorate pulmonary hypertension by blocking EndMT (Tsutsumi et al., 2019) and inhibitors of the HGF receptor c-Met (e.g., carbozantinib) are at present intensively explored in Ewing sarcoma and osteosarcoma clinical trials (Italiano et al., 2020).

As TGF-β is currently considered as potent inducer of EndMT in cancer, targeting TGF-β could represent a potential therapeutic approach. Several TGF-β inhibitors have emerged, among which the small molecule inhibitor galunisertib (LY2157299) appears as one of the most advanced and promising molecules tested in two phase-II trials in pancreatic cancer (NCT01373164) or in hepatocellular carcinoma (NCT01246986). Despite not tested on tumor EndMT yet, galunisertib harbors a very safe toxicity profile, with no cardiac toxicity that was a limitation for the use of the first generation of TGF-β inhibitors tested in clinic (Kovacs et al., 2015). Monoclonal antibodies against TGF-β are now also available and tested in different pathologies. As such, fresolimumab, initially developed for the treatment of idiopathic pulmonary fibrosis, has been investigated in phase-II trials in renal cell carcinoma and melanoma, but also metastatic breast cancer (NCT01401062) (Formenti et al., 2019) where TGF-β blocking was also associated with an increase of the immune anti-tumor response. If the beneficial properties of fresolimumab were confirmed in impairing the process of EndMT, inhibiting the TGF-β pathway could be particularly efficient for the treatment of aggressive tumors with the targeting of two essential components of the TME.

## Conclusion

Despite its beneficial role in embryo development, the process of EndMT may be also detrimental, notably in cancer where it contributes to tumor development. This induced plasticity of the endothelium has an important role in shaping the TME. Endothelial-to-mesenchymal transition participates to initial angiogenesis sprouting, reinforces the stromal fibroblastic microenvironment and remodels the vasculature to support tumor cell dissemination and metastasis. Moreover, it contributes to the resistance to cancer treatment, by affecting directly or indirectly the microenvironment. Thus, the process of EndMT regulates different stages of tumorigenesis from initiation, dissemination to response to treatments.

Exploring EndMT remains very challenging mainly due to its inherent dynamics and to the complexity of its mechanisms. Nevertheless, its full understanding still requires deep exploration. Importantly, the role of EndMT transitional populations still need to be grasped, as these intermediate populations could also be relevant in tumor biology.

In conclusion, EndMT can be considered as an important process that reinforces the TME plasticity and devotion to cancer cells. Therefore, modulation of EndMT could be envisaged as a complementary therapeutic arm to counteract tumor progression. Currently, several strategies to inhibit EndMT are considered and deserve exploration in cancer context.

## Author Contributions

NC, SR, and IC contributed to the writing and editing of the manuscript. SR and IC elaborated the figure and table. All authors contributed to the article and approved the submitted version.

## Conflict of Interest

The authors declare that the research was conducted in the absence of any commercial or financial relationships that could be construed as a potential conflict of interest.
